# Impact of endoscopic ultrasonography with fine needle aspiration assessing clinical lymph node staging on radiotherapy treatment planning in esophageal cancer patients

**DOI:** 10.1093/dote/doaf065

**Published:** 2025-08-07

**Authors:** R B den Boer, M E Sanders, G J Meijer, N Haj Mohammad, M A M T Verhagen, J E Freund, L A A Brosens, B L A W Weusten, P Friederich, L Alvarez Herrero, J P Ruurda, R van Hillegersberg, S Mook

**Affiliations:** Department of Radiation Oncology, University Medical Center Utrecht, Utrecht, The Netherlands; Department of Surgery, University Medical Center Utrecht, Utrecht, The Netherlands; Department of Radiation Oncology, University Medical Center Utrecht, Utrecht, The Netherlands; Department of Surgery, University Medical Center Utrecht, Utrecht, The Netherlands; Department of Radiation Oncology, University Medical Center Utrecht, Utrecht, The Netherlands; Department of Medical Oncology, University Medical Center Utrecht, Utrecht, The Netherlands; Department of Gastroenterology, Diakonessenhuis Hospital Utrecht, Utrecht, The Netherlands; Department of Pathology, University Medical Center Utrecht, Utrecht, The Netherlands; Department of Pathology, University Medical Center Utrecht, Utrecht, The Netherlands; Department of Gastroenterology, University Medical Center Utrecht, Utrecht, The Netherlands; Department of Gastroenterology, Meander Medical Center, Amersfoort, The Netherlands; Department of Gastroenterology, St. Antonius Hospital, Nieuwegein, The Netherlands; Department of Surgery, University Medical Center Utrecht, Utrecht, The Netherlands; Department of Surgery, University Medical Center Utrecht, Utrecht, The Netherlands; Department of Radiation Oncology, University Medical Center Utrecht, Utrecht, The Netherlands

**Keywords:** cancer staging, endoscopic ultrasonography, esophageal cancer, imaging, lymphadenectomy, neoadjuvant chemoradiation, radiotherapy, upper gastrointestinal surgery

## Abstract

Endoscopic ultrasound (EUS) combined with fine needle aspiration (FNA) can be of additional value to fluorine-18 labeled fluorodeoxyglucose positron emission tomography computed tomography (^18^FDG-PET-CT) for lymph node staging in esophageal cancer patients. The study objective was to evaluate the impact of routine EUS-FNA after ^18^FDG-PET-CT staging on radiotherapy planning. Patients with biopsy-proven esophageal carcinoma staged ≥cT2 and eligible for treatment with curative intent, including neoadjuvant chemoradiotherapy (nCRT) or definitive chemoradiotherapy (dCRT), were included. After March 2018, patients who were scheduled for dCRT or ASA 3 were excluded from routine EUS-FNA. The primary outcome was the impact of EUS-FNA after ^18^FDG-PET-CT on radiotherapy target volume delineation. Subsequently, radiotherapy field modifications were compared with surgical pathology when available. Between 2018 and 2023, 179 patients were included. In 61 patients (34%), the EUS scope was unable to pass through the tumor, limiting lymph node assessment. EUS-FNA altered radiotherapy treatment plans in 24 patients (13%), resulting in a number needed to treat of 7.5. Modifications included expansion of the radiation field in 17 cases, reduction in 6 cases, and both in 1 case. Among surgically resected patients, 10 lymph node stations were added to the radiation field based on EUS-FUNA results. Of these, 7 stations (70%) showed no positive or responsive lymph nodes in the resection specimen, while 3 stations (30%) had 2 positive nodes, and 1 with a complete response to nCRT. Four lymph node stations were with no positive nodes found in the resection specimen. Two patients were readmitted post-procedure, including one fatal case of mediastinitis potentially linked to EUS-FNA. Routine EUS-FNA after^18^FDG-PET-CT altered radiotherapy plans in only 13% of patients, with limited and uncertain impact on clinical outcomes, especially for those undergoing planned neoadjuvant therapy and surgery. These findings suggest that EUS-FNA may be best avoided in routine practice for such patients.

## INTRODUCTION

Esophageal cancer is the sixth leading cause of cancer-related death worldwide.^1^ Neoadjuvant chemoradiotherapy (nCRT) followed by surgical resection is the cornerstone of curative treatment.^2^ Accurate staging of esophageal cancer is important for the selection of appropriate treatment strategies for the individual patient. More specifically, staging of metastatic lymph nodes is needed for adequate radiotherapy target planning to maximize dose on the tumor and metastatic lymph nodes. With specific information of the localization of metastatic lymph nodes, surrounding organs at risk could be spared to limit toxicity.

Currently, the standard approach for identifying lymph node metastases is positron emission tomography with computed tomography using fluorine-18 labeled fluorodeoxyglucose (^18^FDG-PET-CT). Endoscopic ultrasonography (EUS) with fine needle aspiration (FNA) is applied increasingly for the diagnosis and staging of esophageal cancer, but its impact on treatment by detecting lymph node metastasis remains uncertain.^3–5^

EUS is a minimally invasive imaging modality that combines endoscopy and ultrasound to provide detailed images of the esophageal wall, the stomach, and the surrounding lymph nodes. FNA can be performed during EUS to obtain cells from suspected lymph nodes for cytologic diagnosis. EUS-FNA-based identification of regional lymph node metastasis can lead to treatment adaptations.^5^ However, EUS-FNA also has a number of limitations, including the discomfort for patients and possible complications, including aspiration, bleeding, and esophageal perforation in less than 1%.^6^^,^^7^ Moreover, tumor-related stenosis of the esophagus hinders complete EUS investigation in one in four patients.^5^

The main objective of this retrospective study was to evaluate the impact of EUS-FNA after ^18^FDG-PET-CT on radiotherapy target volume definition. Radiotherapy field modifications were compared with pathology results of the resection specimen.

## METHODS

### Patient population

A retrospective cohort study was conducted and approved by the institutional review board (number 13/061). The study included consecutive patients with histologically confirmed primary adeno or squamous cell esophageal carcinoma treated with curative intent to undergo multimodality treatment, including preoperative CROSS (chemoradiotherapy for esophageal cancer followed by surgery study) and esophagectomy or definitive chemoradiotherapy (dCRT). Patients were included in University Medical Center Utrecht (UMC Utrecht), a tertiary care center in the Netherlands, between January 2018 and February 2023. EUS-FNA procedures were conducted at either UMCU or two referring hospitals (St. Antonius Hospital Nieuwegein and Meander Medical Center Amersfoort) in the comprehensive cancer network. Following March 2018, patients scheduled for dCRT because of cT4b staged tumor or an American Society of Anesthesiologists (ASA) score of 3 were excluded to prevent severe complications. This amendment of the protocol followed the analysis of a fatal mediastinitis in a patient with a cT3 tumor that was possibly associated with the EUS-FNA procedure.

### Staging process

Patient staging was conducted following the guidelines of the 8th TNM classification by the American Joint Committee on Cancer (AJCC).^8^ This comprehensive staging involved a diagnostic esophagogastroduodenoscopy and tumor biopsy, diagnostic CT scans of the thorax and abdomen, ^18^FDG-PET-CT imaging, and EUS with, if indicated, FNA. The outcomes of all the staging methods were discussed within a multidisciplinary tumor board.

On ^18^FDG-PET-CT scan, lymph nodes were deemed suspicious for metastasis based on evaluation by experienced nuclear medicine physicians and radiologists. These evaluations considered lymph node size, increased uptake of FDG, and lymphatic drainage patterns, allowing distinguishing between malignant and benign disease. During EUS, the following criteria were used to identify suspected metastatic lymph nodes for FNA: a short-axis diameter >5 mm and one or more of the following characteristics: a round shape, a hypoechoic internal aspect, and a distinct border. No FNA was performed if a suspected lymph node was located within 2 cm of the primary tumor, since this lymph node would already be included in the radiation field. EUS findings were performed by independent, experienced endoscopists. Cytological analysis involved identifying metastatic tumor cells that correlated with the primary esophageal tumor.

### Treatment

**Fig. 1:**
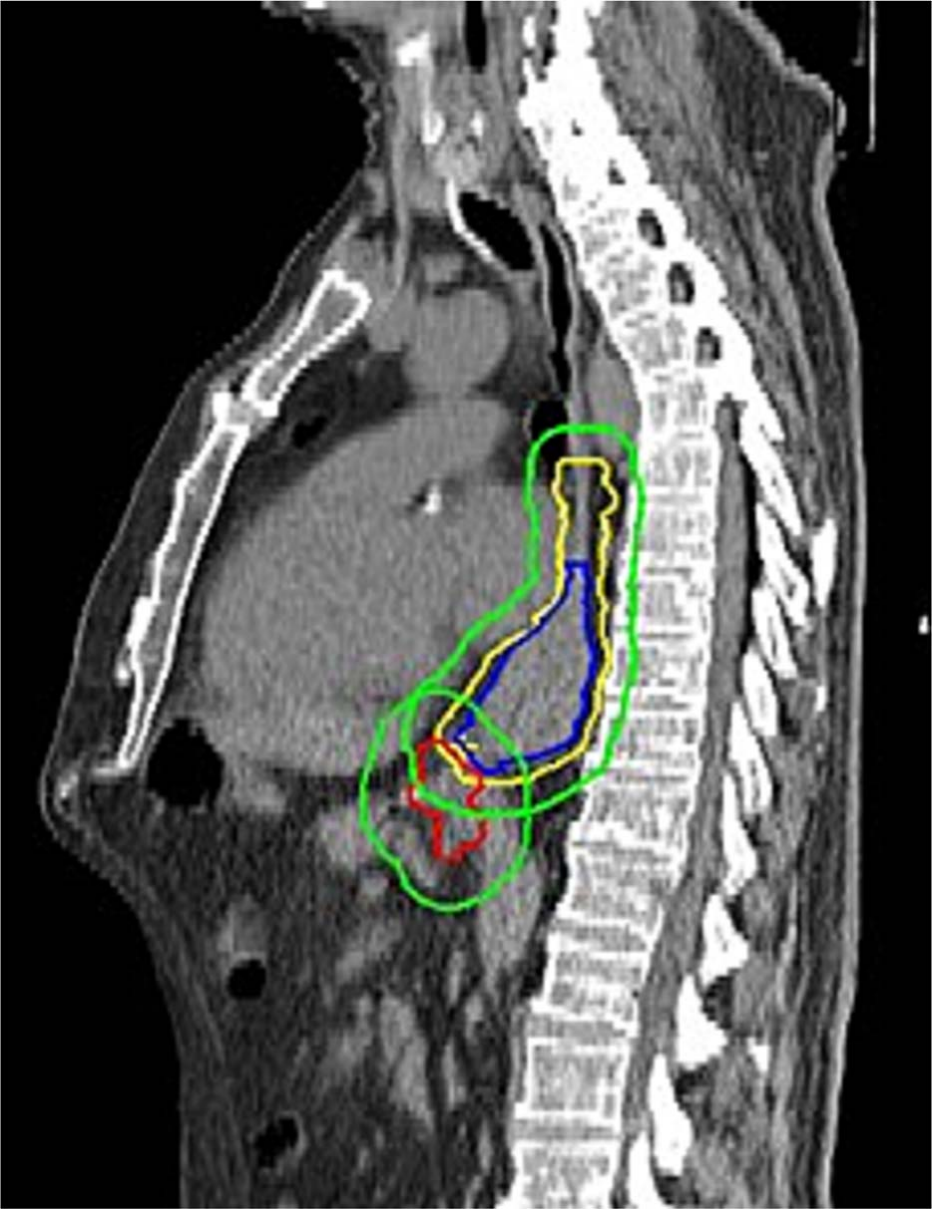
Sagittal slice of a CT scan with an example of a radiotherapy treatment plan involving delineation of the primary tumor (blue = CTV, yellow = GTV, and green = PTV) and separately metastatic abdominal lymph nodes (red).

nCRT involved the administration of 5 times weekly carboplatin area under the curve of 2 mg/mL/min and paclitaxel 50 mg/m^2^ body-surface area according to the CROSS regimen, combined with radiotherapy at a dose of 41.4 Gy delivered over 23 fractions of 1.8 Gy, daily, 5 days a week.^2^ For patients receiving dCRT, the regimen included 6 times weekly carboplatin/paclitaxel with concurrent radiotherapy at a total dosage of 50.4 Gy. In all patients who underwent radiotherapy, a planning CT scan was performed to delineate the gross tumor volume (GTV), considering supplementary staging information. The GTV involved both the primary tumor and suspected metastatic lymph nodes (Fig. 1). The clinical target volume (CTV) was defined by an extension of 3 cm of the GTV of the primary tumor in the craniocaudal directions along the esophageal tract or by 2 cm in caudal direction in cases where the CTV extended in the stomach, as well as a 0.5 cm circumferential margin. The CTV of metastatic lymph nodes was defined as the lymph nodes expanded with of an isotropic margin of 0.5 cm. In the scenario of dCRT, the peri-esophageal fat was included within the radiation field, as surgical resection after dCRT was not expected. Adjustments to the CTV were made to respect the anatomical boundaries of the surrounding organs. The planning target volume (PTV) margin was defined as the total CTV expanded with a 1 cm isotropic margin.

Surgery consisted of robot-assisted minimally invasive esophagectomy (RAMIE) with a two-field lymph node dissection performed within the abdomen and thorax or transhiatal esophagectomy with a limited lymph node resection (abdominal and lower mediastinal nodes). During transthoracic esophagectomy, stations 4L, 4R, 7, 8, and 9 were resected routinely. Stations 2L and 2R were resected only when there was evidence of lymph node metastasis on preoperative imaging, to limit the risk of recurrent nerve palsy. During the abdominal phase, according to the Japanese Classification of Esophageal Cancer 11th edition, lymph node stations 1, 2, 3, 7, 8, 9, and 11 were routinely resected.^9^

In case of cervical lymph node metastasis, a cervical lymph node dissection was performed in selected cases. The decision to perform a transhiatal esophagectomy was based on the patient’s cardiopulmonary comorbidities, as well as the absence of upper mediastinal lymph node metastases. All the surgeries were performed by two experienced gastro-intestinal surgeons (JR and RH, >200 and > 400 RAMIE cases experience, respectively).

### Pathology

During transthoracic esophagectomy, the paratracheal lymph node stations 2L (if indicated) and 4L were sent in separately for histopathologic evaluation, as well as the combination of these lymph node stations on the right. The subcarinal, lower para-esophageal, and gastric lymph node stations were resected en-bloc with the resection specimen of the primary tumor. During transhiatal esophagectomy, the para-esophageal and gastric lymph node stations were resected en-bloc with the resection specimen and the paratracheal and subcarinal lymph node stations could not be dissected. Following resection, lymph node stations were carefully isolated by the surgeon and submitted individually for histopathological evaluation. Histopathological assessment was performed according to standardized clinical protocols by dedicated gastrointestinal pathologists (LAAB, JEF), including assessment of response to neoadjuvant therapy in the irradiated lymph node stations.

### Definition of impact on treatment plan

EUS was considered to have impacted radiotherapy treatment in addition to ^18^FDG-PET-CT if it led to modifications in radiation target volumes, such as the extension or reduction of lymph node stations. An evaluation of EUS’s impact was conducted across the entire patient cohort.

A retrospective assessment of radiotherapy tumor volume delineations was performed by a medical PhD candidate (RB) under the direct supervision of an experienced radiation oncologist (SM). This evaluation focused on determining whether findings from EUS-FNA led to adjustments in radiotherapy target volumes. Guidelines for categorizing lymph node metastasis with conclusive or inconclusive FNA are presented in Tables 1 and 2.

**Table 1:** Guidelines for lymph node metastasis. Guidelines for categorizing lymph node metastasis—in case of conclusive FNA

PET/CT	EUS	FNA	Consider lymph node
+	+	Tumor positive	Metastatic
+	−	Tumor positive	Metastatic
−	+	Tumor positive	Metastatic
−	−	Tumor positive	Metastatic
+	+	Tumor negative	Non-metastatic
+	−	Tumor negative	Non-metastatic
−	+	Tumor negative	Non-metastatic
−	−	Tumor negative	Non-metastatic

**Table 2:** Guidelines for categorizing lymph node metastasis—in case of inconclusive FNA

PET/CT	EUS	FNA	Consider lymph node
+	+	Inconclusive	Metastatic
+	−	Inconclusive	Metastatic
−	+	Inconclusive	Non-metastatic

### Data collection

Patient demographic data, clinical characteristics, EUS findings, histologic diagnosis, radiotherapy planning, and surgical treatment details were extracted from a prospectively maintained database and electronic medical records.

### Outcome measures

The primary outcome was the impact of EUS with FNA on neoadjuvant radiotherapy planning. Secondarily, the modifications based on EUS-FNA were compared to pathology examination of the lymph node stations of the resection specimen. Both tumor-positive lymph nodes as well as lymph nodes with therapy response on chemoradiotherapy were deemed positive.

### Statistical analysis

Descriptive statistics were employed to summarize patient demographics, clinical characteristics, the findings of EUS and FNA, as well as the impact of EUS-FNA on radiotherapy treatment plans. Fisher’s exact tests were used on categorical clinical staging data to identify factors predictive of impact of EUS-FNA on treatment plans. The binary outcome: whether EUS-FNA had an impact or not, was assessed, with factors with a statistically significant *P*-value of less than 0.05 considered statistically significant predictors. All statistical analyses were performed using SPSS statistical software, version 22 (SPSS Inc., Chicago, IL).

## RESULTS

### Patient characteristics

**Fig. 2:**
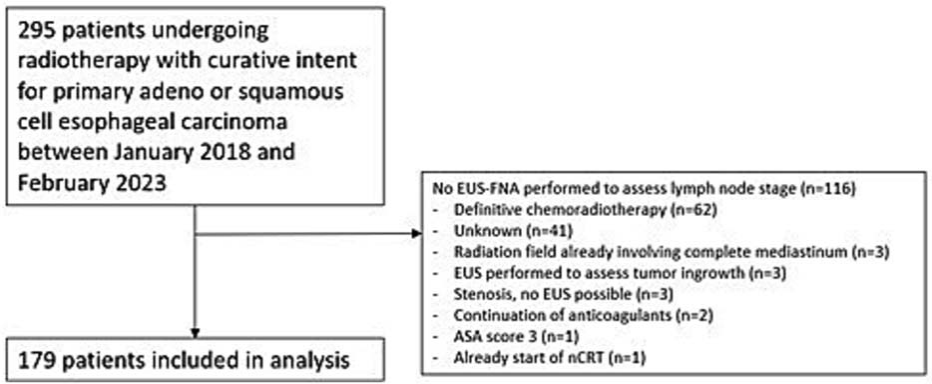
Flowchart of patients included in the study.

A total of 295 patients who underwent radiotherapy for primary esophageal adeno- or squamous cell carcinoma were identified (Fig. 2). 116 patients did not undergo EUS-FNA for a number of reasons, including scheduled for dCRT for a T4b or high esophageal cancer (*n* = 62), and radiation field already involving complete mediastinum due to the extent of the primary tumor (*n* = 3). In 41 patients, EUS-FNA was not performed. In the vast majority of these patients, this was due to altered regulations during the COVID pandemic, in which patient contact was reduced. From the 179 remaining patients, EUS-FNA data assessing lymph node stage was accessible.

**Table 3:** Patient characteristics of patients included in the EUS-FNA analysis

	All included patients undergoing EUS-FNA for assessment of metastatic lymph nodes (*n* = 179)
Male gender	133 (74%)
Age in years; mean (SD)	68 (8.1)
Tumor histology	
Adenocarcinoma	134 (74%)
Squamous cell carcinoma	40 (22%)
Other	5 (3%)
Tumor location	
Upper third esophagus	3 (2%)
Middle third esophagus	16 (9%)
Lower third esophagus	146 (82%)
Gastroesophageal junction	14 (8%)
cT-stage	
T2	38 (21%)
T3	125 (70%)
T4a	8 (4%)
T4b	7 (4%)
Missing	1 (1%)
cN-stage	
N0	87 (49%)
N1	70 (39%)
N2	21 (12%)
N3	1 (1%)
cM-stage	
M0	171 (96%)
M1	1 (1%)
MX	7 (3%)
Treatment	
Definitive chemoradiotherapy	28 (16%)
Neoadjuvant chemoradiotherapy	151 (84%)
Surgical approach	
No surgery	54 (30%)
Transhiatal esophagectomy	18 (10%)
Transthoracic esophagectomy	107 (60%)
EUS	
Complete EUS assessment	118 (66%)
Incomplete EUS assessment, limited by stenosis	61 (34%)

A summary of patient characteristics is presented in Table 3. The mean age of the patients was 68 years, with a majority being male (*n* = 133, 74%). The most common histologic type was adenocarcinoma (*n* = 134, 74%), followed by squamous cell carcinoma (*n* = 40, 22%). Most patients had cT3 tumors (*n* = 125, 70%), with positive lymph nodes based on ^18^FDG-PET-CT in 90 patients (52%). Among the cohort, 151 patients (84%) underwent nCRT, while 28 patients (16%) received dCRT. Surgical resection was conducted in 125 patients (70%), with the transthoracic approach being the most commonly employed method (*n* = 107, 86% of those who underwent surgery).

### Impact of EUS and FNA on radiotherapy plans

In 118 patients (66% of the total), a complete EUS assessment was performed. In the remaining 61 patients (34%), this assessment was hindered by esophageal stenosis, preventing the scope from passing beyond the tumor. A total of 230 mediastinal lymph nodes in 101 patients were considered suspicious for metastasis based solely on EUS assessment. In 92 instances, FNA was omitted as the lymph nodes were located within 2 cm of the tumors. Other reasons to omit FNA are presented in Table 4. In total, FNA was executed 101 times. FNA yielded tumor-positive cytology in 33 lymph nodes (33%), tumor-negative cytology in 61 lymph nodes (60%), and inconclusive outcomes in 7 lymph nodes (7%).

Table 5 displays the effect of EUS-FNA on radiotherapy treatment plans. EUS findings led to modifications of the radiotherapy treatment plan for 24 patients (13%). These modifications included an expansion of the radiation field in 17 cases and reduction in 6 cases. In one instance, the radiation field was simultaneously enlarged as well as reduced with one specific lymph node station, as a direct consequence of the EUS-FNA. Two of the 24 patients had their radiotherapy plans expanded due to the detection of intramural metastasis, diagnosed during EUS.

Of the 24 cases of radiotherapy adjustments, 15 (62%) adjustments could be compared with surgical pathology. 5 patients did not wish to undergo surgery, 1 patient died prior to the operation, 1 patient developed pulmonary interval metastases, 1 patient had a reduction of mediastinal lymph nodes in the radiation field and underwent transhiatal esophagectomy, 1 patient had a cervical lymph node added to the radiotherapy field, which station was ultimately not resected.

Among the patients who underwent surgical resection, where radiation field modifications could be compared with surgical pathology, a total of 10 lymph node stations were added to the radiation field based on EUS-FNA findings. Of these, 7 stations (70%) did not contain metastatic or treatment-responsive lymph nodes in the corresponding resection specimens, suggesting that these additions may not have contributed to oncological benefit. In contrast, 3 stations (30%) contained either metastatic lymph nodes (*n* = 2) or lymph nodes with a complete response to nCRT (*n* = 1), indicating that some modifications aligned with true disease burden. A total of 4 lymph node stations were excluded from the radiation field based on EUS-FNA results. No positive lymph nodes were found in these stations of the resection specimen. Additionally, no clinical staging parameters were significantly associated with treatment alterations, as demonstrated in Supplementary Table S1.

**Table 4:** Fine needle aspiration data

	Total FNA performed (*n* = 101)
FNA (per lymph node)	
Positive	33 (33%)
Negative	61 (60%)
Inconclusive	7 (7%)
	Total lymph nodes (230)
Reasons to omit FNA in EUS-positive lymph nodes (per lymph node)	
Suspected lymph node <2 cm of tumor	92 (40%)
Performance of FNA of another lymph node in the same lymph node station	28 (12%)
Unclear reason	6 (3%)
Continuation of anticoagulants	2 (1%)
Patient discomfort	1 (0%)
Performance of FNA in EUS non-suspicious lymph nodes	8 (3%)

**Table 5:** Radiotherapy treatment consequences of endoscopic ultrasound with or without fine needle aspiration

Treatment consequence	Total cohort (*n* = 179)
Adjustment of radiotherapy field	24 (13%)
Smaller	6 (3%)
Larger	17 (9%)
Both	1 (1%)

### Complications after EUS-FNA

Among the cohort of 179 patients, two individuals required readmission after the EUS-FNA procedure, potentially associated with this intervention. One patient from this group experienced the development of mediastinitis and died, potentially linked to the EUS-FNA procedure.

## DISCUSSION

Esophageal cancer frequently metastasizes to regional lymph nodes, making accurate staging essential for treatment planning, especially in radiotherapy. EUS with or without FNA has been increasingly used for the diagnosis and staging of esophageal cancer, including the detection of lymph node metastasis. This retrospective study evaluated the impact of EUS-FNA in addition to ^18^FDG-PET-CT on radiotherapy treatment planning for esophageal cancer.

In our patient cohort, routine use of EUS-FNA after ^18^FDG-PET-CT scans led to modifications in the CTV in a limited proportion of 13% of the patients, with the most common change an expansion of the radiation field (9%). The rate of non-passable EUS-FNA procedures was relatively high at 34%, resulting in incomplete EUS assessments for a substantial portion of patients. Pathologic lymph nodes on EUS were situated within 2 cm of the primary tumor in 92 lymph nodes (40%), making FNA unnecessary since these nodes were already within the CTV of the primary tumor. Additionally, two patients (1%) required hospitalization due to complications following EUS-FNA, and one of them died from mediastinitis, potentially related to the performance of the EUS-FNA.

Achieving comprehensive validation of EUS-FNA and ^18^FDG-PET-CT with pathology reports poses a significant challenge. The correlation of each lymph node station with its corresponding pathology is complicated by minor discrepancies in the reported stations and the substantial interval between ^18^FDG-PET-CT or EUS-FNA assessment and surgical resection. Moreover, there is a possibility of disease progression between clinical staging and surgical pathology results. It is important to note that changes based on EUS-FNA in radiotherapy plans could be compared in only 55% of cases by the presence of metastatic or responding lymph nodes in that specific part of the resection specimen. Therefore, complete assessment of diagnostic value of EUS-FNA and ^18^FDG-PET-CT for lymph node metastasis could not be performed in this study design.

This study reinforces the acknowledged limitations of ^18^FDG-PET-CT in accurately staging lymph nodes in esophageal cancer.^10^ EUS-FNA has demonstrated value by identifying additional metastatic lymph nodes or intramural metastases, prompting modifications in the radiotherapy plan for 13% of cases. However, with a number needed to treat (NNT) of 7.5, approximately 8 patients require EUS-FNA for 1 to benefit from an adjusted radiotherapy approach, suggesting a relatively high threshold for intervention. While this indicates some utility, the procedure’s immediate impact on treatment decisions may be limited, especially considering that the clinical relevance of altering the radiotherapy plan remains uncertain. While the potential influence of disease progression cannot be entirely dismissed, the superior diagnostic performance of EUS-FNA for lymph node staging is likely to be explanatory. However, the clinical relevance of these findings in terms of patient outcomes, especially in the context of neoadjuvant therapy followed by esophagectomy with an extensive lymphadenectomy, is likely to be minimal. Moreover, the observational design of the current study limits the ultimate impact on clinical outcomes.

Interestingly, radiotherapy demonstrates to have a limited impact on metastatic lymph nodes compared with the primary tumor in esophageal cancer.^11^^,^^12^ This phenomenon is also observed after nCRT in ypT0 esophageal cancer patients, in which presenting positive lymph nodes within the radiation field are found in 15% of cases.^13^ This suggests that precise lymph nodes staging may not be strictly necessary if nCRT is followed by radical surgery and lymphadenectomy. It is also known that regardless of tumor location in esophageal cancer, metastatic lymph nodes can be present in cervical, thoracic, and abdominal regions.^14^ Furthermore, the number of resected lymph nodes has shown an association with overall survival.^15^ Therefore, surgical resection and lymphadenectomy remain crucial in current curative treatment paradigms.

Notably, patients scheduled for dCRT, although predominantly excluded in this cohort, may benefit more from EUS, since they are not anticipated to undergo esophagectomy with extended lymphadenectomy. An Asian randomized trial showed improved overall survival for prophylactic regional nodal irradiation compared with conventional irradiation of enlarged lymph nodes in esophageal squamous cell carcinoma.^16^ The exact role of EUS and potential benefits in this patient group need further investigation. Still, the risk of perforations due to EUS-FNA exists and careful patient selection is important, considering comorbidities and the presence of tumor stenosis.

Previous studies have also explored the impact of EUS on treatment decision-making. The most comparable study found a higher proportion of alterations in treatment decisions (29% treatment alterations and 22% radiotherapy alterations) following EUS-FNA evaluation.^5^ Comparisons with ^18^FDG-PET-CT focused solely on treatment decision influence without comparison with surgical pathology. A systematic review that summarized the diagnostic accuracy of EUS-FNA to detect metastatic lymph nodes included studies where EUS (without FNA) was performed close to surgical resection, yet did not consider additive value to ^18^FDG-PET-CT.^17^ This review reported a pooled sensitivity of 0.62 (95% confidence interval (CI): 0.50–0.73) and specificity of 0.80 (95%CI: 0.73–0.86) for EUS-FNA, indicating that EUS demonstrates moderate to good diagnostic performance for metastatic lymph node detection.

EUS-FNA has known limitations in accuracy for detecting lymph node metastasis, influenced by factors such as operator experience, the location and accessibility of lymph nodes, and node size. Notably, of the 10 lymph node stations identified for expansion of the radiation field, 7 did not demonstrate any metastatic involvement or response in the resection specimens. This discrepancy may stem from difficulties in precisely matching the lymph node stations identified by PET and EUS to those observed in the surgically resected specimen, given anatomical and interpretative variations across these methods. Another potential reason for the discrepancy is the classification of metastatic lymph nodes. In cases where both PET/CT and EUS identified lymph nodes as metastatic, or where only PET/CT identified the nodes as metastatic but the FNA results were inconclusive, the nodes were still classified as metastatic. This could explain why 7 out of the 10 lymph node stations added for radiation field expansion did not show metastasis in the resection specimens. These inconsistencies are likely due to inconclusive FNA results and the inherent limitations of imaging modalities in precisely identifying metastatic disease.

Additionally, EUS and FNA pose risks, including bleeding and, in rare cases, severe complications like perforation.^6^^,^^7^ Thus, EUS use in radiotherapy planning should be individualized, with clearer benefits in patients not undergoing radical surgery and lymphadenectomy. However, this study did not identify any specific subgroup with a higher likelihood of treatment alteration based on the available clinical data.

The ongoing debate regarding the optimal neoadjuvant therapy for esophageal adenocarcinoma, the most prevalent histological subtype in the Western world, may also impact the importance of lymph node staging in esophageal cancer. Increasing attention is being directed toward neoadjuvant chemotherapy followed by surgery for adenocarcinoma.^18^ The forthcoming ESOPEC trial is anticipated to offer insights into this matter, randomizing patients between neoadjuvant chemoradiotherapy and perioperative chemotherapy.^19^ Concurrently, addition of immunotherapy in the neoadjuvant setting is an ongoing field of research.^20^ Proton therapy holds promise in reducing the toxicity associated with neoadjuvant radiotherapy, although its precise indications are still subject to ongoing debate.^21^ This shift in focus may affect the relevance of achieving optimal lymph node staging or even the need for radical lymphadenectomies in the context of esophageal cancer in the future.

Beyond its application in lymph node diagnosis, EUS can play a role in determining the T-stage of esophageal cancer. If PET-CT scans raise concerns regarding potential aortic involvement, performing an EUS after dCRT provides an option for reassessing tumor ingrowth. This evaluation not only assesses treatment response but also identifies patients who could benefit from a potentially curative salvage esophagectomy.^22^ While EUS’s use in this context is acknowledged, this was not addressed within the current study.

Strength of this study included its thorough evaluation of treatment implications within a Western patient cohort of mainly adenocarcinomas of the esophagus. However, limitations arose due to hindered comparison with pathology results and the inability to directly assess the impact on actual patient outcomes.

Based on this retrospective cohort study, routine use of EUS (+/− FNA) in addition to 18FDG-PET-CT to evaluate lymph node metastases results in change of the radiotherapy treatment in 13% of the patients. This modest rate of adaptation, paired with an NNT of 7.5, underscores the questionable value of EUS-FNA in this setting, especially when it remains uncertain if these treatment modifications provide a meaningful clinical benefit. Given these findings, EUS-FNA may be best avoided in routine practice for patients scheduled for neoadjuvant therapy followed by surgery. Instead, its use might be limited to cases where surgical intervention is not part of the treatment plan and where lymph node staging directly influences the primary therapeutic approach.

## Supplementary Material

Supplementary_Table_1_v24_doaf065

## Notes

Specific author contributions: Robin Den Boer (Conceptualization, Data curation, Formal analysis, Investigation, Methodology, Project administration, Validation, Writing—original draft, Writing—review & editing), Maxime Sanders (Conceptualization, Data curation, Formal analysis, Investigation, Methodology, Project administration, Validation, Visualization, Writing—original draft, Writing—review & editing), Gert Meijer (Conceptualization, Investigation, Supervision, Writing—review & editing), Nadia Haj Mohammad (Investigation, Supervision, Writing—review & editing), Marc Verhagen (Investigation, Writing—review & editing), Jan Erik Freund (Investigation, Writing—review & editing), Lodewijk AA Brosens (Investigation, Writing—review & editing), Bas L Weusten (Conceptualization, Formal analysis, Investigation, Writing—review & editing), Philip Friederich (Investigation, Writing—review & editing), Lorenza Alvarez Herrero (Investigation, Writing—review & editing), Jelle Ruurda (Formal analysis, Investigation, Supervision, Writing—review & editing), Richard van Hillegersberg (Formal analysis, Investigation, Supervision, Writing—review & editing), Stella Mook (Conceptualization, Data curation, Formal analysis, Investigation, Methodology, Project administration, Supervision, Validation, Writing—original draft, Writing—review & editing).

Financial support: No financial support was received nor requested for the research, authorship, and/or publication of this article.
