# Supplementary material for: Impact of endoscopic ultrasonography with fine needle aspiration assessing clinical lymph node staging on radiotherapy treatment planning in esophageal cancer patients
Source: Dis Esophagus. 2025 Aug 7;38(4):doaf065. doi: 10.1093/dote/doaf065 (PMC12342360; doi:10.1093/dote/doaf065)
Supplement: Supplementary_Table_1_v24_doaf065 [file supplementary_table_1_v24_doaf065.docx]

*Supplementary Table 1. Differences in clinical (staging) data between patients undergoing radiotherapy field alterations based on EUS-FNA and those who did not.*

|  | Radiotherapy field alterations (n=24) | No radiotherapy field alterations (n=155) | P-value |
| --- | --- | --- | --- |
| Tumor histology |  |  | 0.143 |
| Adenocarcinoma | 22 (92%) | 112 (72%) |  |
| Squamous cell carcinoma | 2 (8%) | 38 (25%) |  |
| Other | 0 (0%) | 5 (3%) |  |
| Tumor location |  |  | 0.711 |
| Upper third esophagus | 0 (0%) | 3 (2%) |  |
| Middle third esophagus | 2 (8%) | 14 (9%) |  |
| Lower third esophagus | 19 (79%) | 127 (82%) |  |
| Gastroesophageal junctions | 3 (13%) | 11 (7%) |  |
| cT-stage |  |  | 0.732 |
| T2 | 6 (25%) | 32 (21%) |  |
| T3 | 18 (75%) | 107 (70%) |  |
| T4a | 0 (0%) | 8 (5%) |  |
| T4b | 0 (0%) | 7 (5%) |  |
| Missing | 0 | 1 |  |
| cN-stage |  |  | 0.179 |
| N0 | 13 (54%) | 71 (47%) |  |
| N1 | 10 (42%) | 56 (37%) |  |
| N2 | 0 (0%) | 21 (14%) |  |
| N3 | 1 (4%) | 4 (3%) |  |
| Missing | 0 | 3 |  |
